# Reflections from the bedside: a nursing perspective on three decades of intensive care

**DOI:** 10.1186/s13054-023-04767-1

**Published:** 2023-12-07

**Authors:** Kathryn A. Riman, Deena Kelly Costa

**Affiliations:** 1 Department of Critical Care Medicine, University of Pittsburgh School of Medicine, 3550 Terrace Street, Pittsburgh, PA 15261 USA; 2 Yale School of Nursing, Yale University School of Medicine, 400 West Campus Drive, Orange, CT 06477 USA

## Introduction

Drawing from Maurizio Cecconi's editorial, we offer interprofessional insight into critical care's future, emphasizing the interplay of technology and human connection.

## The evolution of critical care nursing

Since critical care’s inception in the 1950s, nursing has been central in its development. Critical care nurses were and remain essential members of the interprofessional team that deliver direct patient care to critically ill patients [1]. Their role has evolved significantly from being primarily task-oriented to encompassing a multifaceted profession characterized by clinical expertise, technological proficiency, unwavering patient advocacy, and compassionate care. The global COVID-19 pandemic further expedited this shift, accelerating the transformation of critical care nursing into a highly specialized and collaborative discipline. Amid the crisis, critical care nurses emerged as emotional support for isolated patients, providing comfort and connection during the most challenging of times, while also rapidly acquiring new skills to master everything from personal protective protocols to ventilation techniques and end-of-life care in unprecedented conditions.

This period also witnessed rapid changes in the conceptualization and operationalization of critical care nursing; there was the strategic redeployment of nurses from various specialties into ICUs, the inclusion of travel nurses, and the expansion of virtual nursing. These adaptations were crucial to address fluctuating patient volumes during the pandemic, underscoring the need for dynamic staffing models that could adapt to changing demands. Virtual nursing emerged as a valuable resource, empowering critical care teams to provide remote monitoring and support to patients across multiple locations. By leveraging telemedicine, virtual critical care nurses could augment the bedside workforce and deliver specialized care to patients in underserved areas. These staffing models, encompassing team nursing, pairing less experienced nurses with seasoned ICU veterans, flexible staffing ratios based on patient acuity, and virtual nursing, became instrumental in delivering quality care during crises. However, they also brought to the forefront the stark realities of current challenges, including workforce shortages and the pressing need for robust support and continuous professional development within the nursing field.

The rapid implementation of new technologies and practices in response to the pandemic highlights the need for a more evidence-based approach to healthcare innovation. While the use of virtual nursing and other staffing models proved beneficial during a crisis, it is essential to conduct rigorous research to assess their long-term effectiveness and determine the optimal implementation strategies. Staff nurses play a crucial role in this process, providing valuable insights from their frontline experience and expertise in patient care. Moreover, nurse researchers can and should be leading the evaluation, testing and implementation of these staffing models. By actively engaging in research and development, nurses can ensure that new technologies and practices are not only effective but also aligned with the core values and principles of the nursing profession.

## Challenges and opportunities in critical care today

The pandemic highlighted the need for dynamic staffing models in critical care, but it also laid bare the underlying challenges of continually expanding nursing skill sets to provide specialized care for increasingly complex patients. The substantial turnover of experienced nurses exacerbates these challenges, often placing less experienced nurses at the forefront of patient care. To address these pressures, critical care environments must adapt by implementing supportive staffing models, facilitating continuous education, and nurturing a culture of mutual respect and teamwork.

While staffing models provide a general framework for assigning nurses to patients, to move forward, critical care must adopt a more multifaceted approach that considers factors beyond the number of patients per nurse; such an approach should consider nurse familiarity and patient acuity. Research has shown that nurse-to-nurse familiarity, defined as sharing more patients with other nurses, and illness severity among nurses’ assignments are both associated with mortality in the ICU [2–4]. By considering these factors, critical care can develop more effective and efficient staffing models that promote positive patient outcomes and pave the way for the next century of critical care.

While nurse-to-patient assignments are essential for ensuring optimal patient care, critical care nurses must also adapt to a dual role in the digital age: integrating technological advancements, such as virtual nursing staffing models, while preserving the core values of their profession. In addition to virtual nursing staffing models, other technological advancements, such as artificial intelligence (AI) algorithms and telemedicine, are also assuming greater significance in critical care nursing [5]. AI algorithms empower nurses to analyze vast quantities of patient data in real time, predict potential complications, and provide personalized care to each patient. Telemedicine further supports critical care nurses by enhancing communication and collaboration among healthcare team members and empowering patients to take a more active role in their care. By embracing technological advancements and thoughtfully integrating them into their practice, critical care nurses can elevate the quality and efficiency of care, ensuring they continue to meet the evolving needs of patients and healthcare systems.

## Vision for the future of critical care nursing

Critical care nursing faces numerous challenges necessitating innovative solutions. The seamless coordination of diverse teams and the intricacies of nurse-to-patient assignments require ongoing research and advancements. Investing in research is pivotal for optimizing ICU environments and refining nursing care to achieve the best possible patient outcomes. More importantly, nurse researchers must be leading the way in designing the future of critical care. Striking the right balance between technology and humanity is essential for revolutionizing critical care. Despite these challenges, we are optimistic about the future of critical care nursing. We firmly believe that nurses will be central to the provision of holistic, patient-centered care. We believe that nurse researchers will continue to lead in determining how best to organize critical care and how best to support the profession. By embracing personalized nurse-to-patient assignments and optimizing interprofessional teams, nurses can continue to deliver truly holistic care, leading to enhanced patient outcomes and improved healthcare delivery.

## Conclusion

In conclusion, critical care nursing's evolution, shaped by the pandemic, necessitates dynamic staffing, technological integration, and a focus on compassionate care. Emphasizing a multifaceted approach and research, the field must balance technology and humanity to continue providing patient-centered care.

## Data Availability

Not applicable.

## References

[1] Fairman J, Lynaugh JE. Critical Care Nursing: A History. University of Pennsylvania Press, Incorporated, https://books.google.com/books?id=gQE69t0cbrMC (2000).

[2] Riman KA, Davis BS, Seaman JB, et al. Association between nurse copatient illness severity and mortality in the ICU. Crit Care Med. 2023;10–1097. 10.1097/CCM.0000000000006066 PMC10840670 37846937

[3] Kelly Costa D, Liu H, Boltey EM (2020). The structure of critical care nursing teams and patient outcomes: a network analysis. Am J Respir Crit Care Med.

[4] Duclos A, Payet C, Baboi L (2023). Nurse-to-nurse familiarity and mortality in the critically ill: a multicenter observational study. Am J Respir Crit Care Med.

[5] Keim-Malpass J, Moorman LP (2021). Nursing and precision predictive analytics monitoring in the acute and intensive care setting: an emerging role for responding to COVID-19 and beyond. Int J Nurs Stud Adv.

